# PETModule: a motif module based approach for enhancer target gene prediction

**DOI:** 10.1038/srep30043

**Published:** 2016-07-20

**Authors:** Changyong Zhao, Xiaoman Li, Haiyan Hu

**Affiliations:** 1 Department of Electrical Engineering & Computer Science, University of Central Florida, Orlando, FL, 32816, USA; 2 Burnett School of Biomedical Science, University of Central Florida, Orlando, FL, 32816, USA

## Abstract

The identification of enhancer-target gene (ETG) pairs is vital for the understanding of gene transcriptional regulation. Experimental approaches such as Hi-C have generated valuable resources of ETG pairs. Several computational methods have also been developed to successfully predict ETG interactions. Despite these progresses, high-throughput experimental approaches are still costly and existing computational approaches are still suboptimal and not easy to apply. Here we developed a motif module based approach called PETModule that predicts ETG pairs. Tested on eight human cell types and two mouse cell types, we showed that a large number of our predictions were supported by Hi-C and/or ChIA-PET experiments. Compared with two recently developed approaches for ETG pair prediction, we shown that PETModule had a much better recall, a similar or better F1 score, and a larger area under the receiver operating characteristic curve. The PETModule tool is freely available at http://hulab.ucf.edu/research/projects/PETModule/.

The identification of enhancer-target gene (ETG) pairs is pivotal to our understanding of gene transcriptional regulation^1^,^2^. Enhancers are short regulatory DNA sequences that enhance the expression levels of their target genes^2^. They can be millions of base pairs (bp) away from target genes and interact with target gene promoters through looping^3^. Enhancers are in general several hundred to several thousand bp long, and often contain binding sites of multiple transcription factors (TFs)^2^,^4^. These multiple TF binding sites (TFBSs) in the same enhancers together with TFBSs in promoters of their target genes modulate the tissue and cell-specific expression of target genes^5^. Because of such important roles of enhancers on gene transcriptional regulation, it is fundamentally important to identify ETG pairs.

It is still challenging to identify ETG pairs. The difficulty lies in the fact that genes hundreds of thousands to millions of bp away from an enhancer can be its target genes^2^. With many genes that may exist in such a long region for a given enhancer, it is nontrivial to distinguish a small number of true targets from a large number of other genes in this region. Moreover, enhancers modulate expression of their targets in a cell- and tissue-specific fashion, which implies that the same enhancer may have different targets under different conditions^2^. In addition, an enhancer may target multiple genes interspersed by non-target genes^6^. To accurately identify ETG pairs, one thus have to pinpoint the true targets from a large number of genes in a condition-specific matter.

Several experimental approaches are available for identifying ETG pairs. The chromosome conformation capture (3C) experiment cross-links two interacting regions such as an enhancer and one of its target promoters and then measures the level of interactions by polymerase chain reactions^7^. The 4C and 5C experiments improve the 3C technique and can more unbiasedly measure the interactions of more regions at one time^8^,^9^. Later, the Hi-C experiment couples these low-throughput techniques with next generation sequencing technologies and can identify genome-wide interacting regions^10^. Early Hi-C experiments have a low resolution of 1 megabase (Mb), which means that interacting regions shorter than 1Mb often cannot be detected^10^,^11^. Recent Hi-C experiments have achieved a 1 or 5 kilobase resolution, with a significant increment of the sequencing depth^12^,^13^. Besides these 3C derivatives, the chromatin interaction analysis by paired-end tag sequencing (ChIA-PET) measures genome-wide interactions anchored by a specific factor^14^. Although the sequencing depth requirement is not high, ChIA-PET cannot detect interacting regions that do not involve the factor under consideration.

A few computational methods have been developed for ETG pair identification. The simplest approach takes the closest gene to an enhancer as its target. Such an approach may generate a large number of false positives^15^. With the flood of high throughput data, several studies calculate the correlation of specific signals in enhancer regions and those in proximal regions around gene transcriptional start sites (TSS) and choose gene(s) with the best correlation as target(s). For instance, Thurman *et al*. calculated the correlation of DNase I hypersensitivity signals (DHSs) measured by DNase I hypersensitive analysis followed by sequencing (DNase-seq) experiments to infer ETG pairs^16^. Shen *et al*. predicted ETG pairs with the correlation of H3K4me1 measured by chromatin immunoprecipitation followed by massive parallel sequencing (ChIP-seq) experiments^17^. Although these correlation based approaches may identify multiple targets of an enhancer, the correlation calculation may be greatly affected by the selected experiments and certain target genes may be missed. For instance, assume an enhancer regulates its target gene A in *n* experiments. Assume this enhancer regulates another target gene B in another set of *n* experiments. If the first n experiments are chosen for the correlation calculation, the target gene B will not be discovered. A few more sophisticated methods than the correlation calculation are also available^18^,^19^,^20^,^21^. For instance, a recent approach called IM-PET achieves an area under the receiver operating characteristic curve (AUC) of 0.89 and a F1 score of 0.3 for ETG pair prediction^19^.

Here we developed an integrated computational approach called PETModule (Predicting enhancer target by modules) for predicting ETG pairs. PETModule integrates the conservation, distance, enhancer-promoter activity correlation used previously^16^,^18^, and a brand-new feature based on motif module into one prediction model. A motif module is defined as a group of DNA binding motifs that significantly co-occur in short genomic regions of 1 kilobase long or so^22^,^23^,^24^. Tested on eight human cell types, we showed that on average, PETModule achieved an AUC of more than 0.94 and a F1 score larger than 0.32. Next, we studied the common characteristics of the predicted ETG pairs and revealed special characteristics of ETG pairs. For instance, targets of the same enhancers were often interspersed with non-target genes of the enhancers. In addition, we investigated the importance of the features used in PETModule and demonstrated that the enhancer-promoter activity correlation feature was the least important one. Without this feature, we applied PETModule to two mouse cell types and showed that it on average had an AUC of 0.93 and a F1 score larger than 0.33. Compared with IM-PET^19^ and PreSTIGE^20^, two recently developed tools for ETG pair prediction, we shown that PETModule had a much better recall, a better AUC and a similar or better F1 score on the human datasets. It is worth mentioning that users need input only the enhancer locations for ETG pair prediction by PETModule, which is much simpler and user-friendly than available methods. The PETModule tool is freely available at http://hulab.ucf.edu/research/projects/PETModule/.

## Results

### Predicted ETG pairs in human cell types were supported by Hi-C and ChIA-PET data

We applied PETModule to predict ETG pairs in eight human cell types (Supplementary data S1). There were Hi-C and/or ChIA-PET data available in four of these eight cell types. Since the Hi-C resolution in these cell types were larger than 5 kilobases, we only considered the predicted ETG pairs that were at least 5 kilobase apart to assess the accuracy of PETModule when the predicted ETG pairs were compared with the ETG pairs from Hi-C. Moreover, we assumed that all ETG pairs from Hi-C or ChIA-PET data were true ETG pairs. For each enhancer, we considered all other genes within 2 Mb to this enhancer that were not identified to interact with this enhancer by Hi-C and ChIA-PET as false ETG pairs. With the true and false ETG pairs other than those used for training (methods), we assessed the predictions by PETModule.

Overall, PETModule had a high recall and a high AUC (Table 1). The recall measured how many percent of known ETG pairs were predicted as target genes of the corresponding enhancers. The AUC, which was between 0 and 1, measured the ability of a predictor such as PETModule to distinguish true ETG pairs from false ones. The closer to 1 the AUC was, the more accurate prediction the predictor made. The lowest recall was 0.397 in IMR90 and the highest recall was 0.505 in MCF7 (Table 1). On average, PETModule had a recall of 0.411, a precision of 0.273, and an AUC larger than 0.94.

The above analyses were based on ETG pairs defined in the published papers^13^,^25^. Alternatively, one may define “true” ETG pairs as pairs that were supported by at least a given number of reads in the normalized Hi-C experiments. Rao *et al*. did Hi-C experiments in eight human cell types, three of which (GM12878, K562, IMR90) we had enhancer data and applied PETModule to. Using the normalized Hi-C contact matrices from Rao *et al*.^12^, we defined ETG pairs with different minimum number of supporting Hi-C reads and assessed the accuracy of PETModule in the three cell types. We summarized the results in Table 2 and the Supplementary Tables S1 and S2.

We found that a much larger number of predicted ETG pairs were supported, compared with the results summarized in Table 1, implying that a large number of predictions by PETModule were likely functional and the precision of PETModule in Table 1 may be underestimated. For instance, the precision was 0.74 in IMR90 (Table 2), when at least 5 supporting Hi-C reads were required. When the minimum number of supporting Hi-C reads increased, the percentage of supported predictions decreased. When more than 25 supporting reads were required, the recall, precision, AUC, and F1 score in IMR90 were similar to what we obtained in Table 1. Note that the IMR90 Hi-C data in Tables 1 and 2 were generated by different labs. The consistency of the performance in IMR90 in the two tables indicated that the experimentally defined target genes we used were reproducible and reliable.

To further assess the significance of a large number of predicted ETG pairs were supported by at least a given number of reads, we generated the same number of random enhancer-gene pairs in each cell type. A random pair was generated by randomly selecting a gene within 1 Mb or 2 Mb neighbourhood of an enhancer. We found that the number of predicted ETG pairs supported by at least a given number of reads was not by chance (Supplementary Tables S3–S5). Instead, these predicted ETG pairs supported by at least a given number of reads were likely functional. For instance, in IMR90, 74.0%, 52.6%, 39.8%, 31.6%, and 26.3% of ETG pairs predicted by PETModule within 2 Mb of the corresponding enhancers were supported by at least 5, 10, 15, 20 and 25 Hi-C reads, respectively; while the corresponding numbers for random pairs were 10.2%, 5.4%, 3.8%, 3.1% and 2.6%, respectively. The chance to observe the above percentages of the predicted ETG pairs supported was 0 in all cases based on binomial tests (Supplementary Tables S6–S8). This indicated that a large number of predicted ETG pairs were likely biologically meaningful and the precision of PETModule estimated in Table 1 were underestimated.

### Predicted ETG pairs reveals new characteristics

With the predicted ETG pairs supported by high-throughput experiments, we studied their characteristics. We found that a large number of target genes may be more than 1 Mb away from their enhancers. We also found that more than 69% of enhancers had their target genes that were not consecutive in the genome. The details were in the following.

We compared the predictions from 1 Mb with those from 2 Mb of enhancers (Supplementary Table S9). We found that the recall of the predictions from 1 Mb of enhancers was smaller than that from 2 Mb of enhancers. This was especially true in K562, where the recall increased 26.8%. The different recalls indicated that a large number of true target genes were more than 1 Mb away from their enhancers. We also noticed that the number of the predicted ETG pairs were doubled and the precision on average decreased about 8.3% when targets were predicted from 2 Mb instead of 1 Mb neighbourhood of enhancers, suggesting that a much higher percentage of genes within 2 Mb were not true targets than that within 1 Mb of enhancers. Moreover, the AUC of the prediction was improved, implying the importance of considering 2 Mb instead of 1 Mb neighbour of enhancers for target identification.

We also investigated the inconsecutiveness of target genes in the genome (Supplementary Table S10). Surprisingly, we found that on average targets of 69.9% of enhancers were not consecutive in the genome. Instead, targets of these enhancers were interspersed with non-targets of the same enhancers. This surprising observation may be explained by the following model: DNA forms loops so that targets of the same enhancer are in proximal regions around enhancers. Because of the loops, certain target genes in a consecutive genomic region are much farther relative to the enhancers than other non-target genes in the same region. This observation suggested that when defining regulatory regions of a gene, one should consider all potential regulatory regions within 2 Mb, even if other genes existed between a regulatory region and the gene under consideration.

### The function similarity score (FSS) feature was more important than the widely used correlation feature

PETModule used the following four features to predict ETG pairs: distance, conserved synteny score (CSS), FSS, and correlation (Methods). To understand the importance of the four features, we applied four approaches to rank them (Methods). The four methods used were information gain attribute evaluator^26^, support vector machines (SVM)^27^, least absolute shrinkage and selection operator (LASSO)^28^ and random forests^29^. These methods showed good performance in selecting or ranking features in previous studies^26^,^30^,^31^,^32^. To rank the four features, we used the 2500 positive ETG pairs and 2500 negative ETG pairs in the training data (Methods).

**Figure 1:**
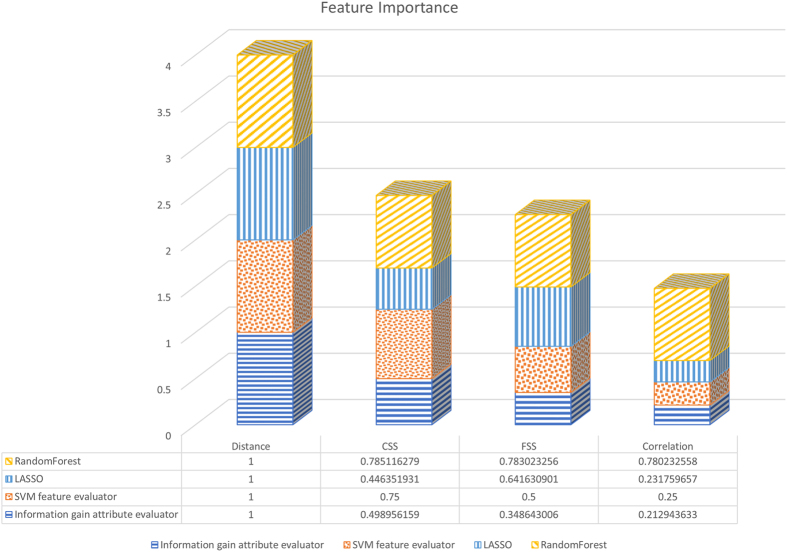
The importance of the four features ranked by four methods.

We found that the four features were ranked similarly by the four methods (Fig. 1). The distance was the most important feature, followed by CSS, FSS, and correlation. The importance of the CSS and FSS features was similar, with CSS slightly more important than FSS based on three methods and FSS more important than CSS based on LASSO. The importance of CSS and FSS was much more than that of correlation, which was ranked as the least important feature by all methods.

The above correlation calculation was based on 13 cell types. To see whether the correlation calculated from another set of properly chosen cell or tissue types may improve the importance of this feature, we further studied the rank of the correlation using the 12 cell types in a previous study^20^. Consistently, the correlation feature was still ranked as the least important feature (Supplementary Figure S1).

The FSS feature was ranked the third by the methods. We hypothesized that the importance of the FSS feature may be underestimated with the current incomplete gene ontology (GO) annotation. We thus tested how the accuracy of PETModule changed if earlier version of GO annotation instead of the current one was used. We found that if we used the GO annotation in 2001, the recall decreased 12.4% while the precision was similar (Supplementary Table S11). This comparison with different GO annotation versions supported the importance of the FSS feature, and suggested that the FSS feature would be even more important with more complete GO annotation in the future.

### Predicted mouse ETG pairs were supported by Hi-C and 3C data

We applied the trained model on the human data without the correlation feature directly to two mouse datasets in the CH12 cell line and the macrophage cell. We found that with such a model trained on the human datasets, the accuracy of PETModule on the two mouse datasets was similar to that in the human datasets (Table 3). For instance, the AUC was 0.938 and 0.923 in CH12 and macrophage, respectively, which were similar to the AUC in the three human cell types in Table 1. We also noticed that the precision and the F1 score of PETModule decreased. PETModule still had a recall of 0.667 and 0.65 in CH12 and macrophage, respectively. The performance of PETModule in CH12 was more similar to that in the three human cell types in Table 1, which may be due to the fact that the supporting Hi-C data in CH12 were generated by the same lab as those in the three human cell types. We believed that the limited number of experimentally validated ETG pairs in mouse macrophage by 3C may partially explain the lower accuracy of PETModule on this mouse dataset than that on the other mouse dataset.

To see whether the model specifically trained on mouse data would perform better than the model trained on the human data, we generated the mouse PETModule with the correlation feature (Methods). We found that the mouse PETModule was much better than the one trained on the human data when applied to the mouse datasets (Table 3). For instance, the trained model using the mouse data had a more than 5% higher recall and more than 9% higher precision. The much improved performance suggested that there existed difference between human ETG pairs and mouse ETG pairs, which may be critical for improving the precision of the computational prediction of ETG pairs in mammalian species.

### PETModule showed good performance when compared with existing tools

We compared PETModule with IM-PET and PreSTIGE^19^,^20^. IM-PET and PreSTIGE each integrated several types of data to predict ETG pairs in human and showed better performance than several other existing approaches^19^,^20^. To compare, we downloaded their predictions in the three cell types for which we applied PETModule and there existed ChIA-PET and/or Hi-C datasets for validation. We found that PETModule had a higher AUC and recall on these datasets than the two methods.

We compared the three tools using the ETG pairs defined by previous studies (Supplementary Table S12). Under the default cutoff, 0.95 (Supplementary Table S13), PETModule had a much higher recall and AUC, a higher precision and F1 score than IM-PET, suggesting it predicted more true ETG pairs and had a higher accuracy. PETModule also had a much higher recall and AUC but a slightly lower precision and F1 score than PreSTIGE. The lower precision and F1 score may be caused by the fact that PETModule predicted ETG pairs within 2 Mb neighbourhood of an enhancer, while PreSTIGE predicted ETG pairs within 100 kb neighbourhood of an enhancer. In fact, if PETModule only considered ETG pairs with 1 Mb to enhancers, its precision were similar or much larger than that of PreSTIGE, even PreSTIGE still predicted much smaller number of ETG pairs (Supplementary Tables S9 and S12). Because the enhancers used by the three tools were different, we further compared their predictions on the common set of enhancers (Table 4). We had similar observation. That was, we found that PETModule had a higher AUC and recall, higher or similar precision and F1 score compared with the other two methods.

We also compared the three methods using the potential ETG pairs supported by at least a given number of supporting Hi-C reads in three cell types (Supplementary Tables S14–S16). Under different cutoffs, PETModule in general had a similar or better performance when compared with IM-PET and PreSTIGE. For instance, in IMR90, when the cutoff was 20, PETModule had a recall of 0.432 and a F1 score of 0.365, while IM-PET and PreSTIGE had a recall of 0.185 and 0.239, respectively, and a F1 score of 0.249 and 0.278, respectively.

We also compared the speed of the two methods (Supplementary Table S17). For the 5000 example enhancers provided in the IM-PET tool, it took IM-PET, PETModule and PreSTIGE 0.5, 5, and 6 CPU hours, respectively, to predict ETG pairs. Note that the time cost mentioned above did not consider the extra time cost required by IM-PET and PreSTIGE to prepare the input files for ETG pair prediction. IM-PET required to run four tools and PreSTIGE required to prepare three different input files before ETG pair prediction. For instance, IM-PET required the running of CSI-ANN to obtain the enhancer signals, which took about 4 CPU hours for 5000 enhances. Therefore, the running time of IM-PET and PreSTIGE was much longer than PETModule in practice.

It is worth mentioning that PETModule is much easier to use. PETModule requires only the enhancer locations as input to predict ETG pairs and utilizes about a dozen pre-selected cell types to calculate the values of the correlation feature. In contrast, IM-PET and PreSTIGE require users to input enhancer positions, genome-wide enhancer signals, and gene expression information, which requires extra experiments done under a condition and extra calculation before any ETG pair prediction. It is thus evident that PETModule provides a much simpler solution for ETG pair prediction in mammals.

## Discussion

We developed a useful ETG pair prediction tool called PETModule. Tested on eight human cell types, we showed that on average PETModule had a recall of 41.1%, a precision of 27.3%, and an AUC of 94.9%. By studying the predicted ETG pairs, we discovered interesting characteristics of ETG pairs. We also compared PETModule with IM-PET and PreSTIGE^19^,^20^, and demonstrated that PETModule had a higher recall, a similar or higher F1 score, and a higher AUC. The PETModule tool is freely available at http://hulab.ucf.edu/research/projects/PETModule/.

A unique feature used in PETModule is the FSS feature, which measures the GO similarity of enhancers and genes. This new feature is different from those used previously^18^,^19^, which use the GO similarity of the targets and the genes encoding TFs that bind the corresponding enhancers. The difference at least lies in the following two aspects. First, the new feature considers groups of regulatory motifs that co-occur in enhancers, while previous studies only consider individual known TFs that bind the enhancers. Second, the new feature considers multiple enhancers and their neighbourhood genes to infer the GO terms of enhancers, while previous studies simply utilize the known GOs of the TFs that bind enhancers and consider individual enhancers at a time. In high eukaryotes, it is often the multiple TFs together instead of individual TFs that determine the temporal and spatial expression patterns of genes, and genes regulated by the same group of TFs often have similar functions or GO terms^22^,^33^,^34^. It is thus more natural to consider a group of co-occurring regulatory motifs in enhancers and take all enhancers with similar motifs into account to infer the potential functions of an individual enhancer. It is worth pointing out that one cannot use the GO terms of the predicted motifs to infer the GO terms of enhancers, as predicted motifs are often new or may be similar to only poorly annotated known motifs.

Our study showed that the correlation of certain signals in enhancers and those in the corresponding targets was not as important as other features in predicting ETG pairs. However, our study also supported that the activity of many enhancers may significantly correlate with that of their targets. The significant correlation may be due to the fact that when enhancers are shared by different tissues or cells, the majority of their ETG pairs are also likely shared (Supplementary Tables S18 and S19). Certainly, there exist a fraction of ETG pairs not shared by different tissues or cell types, even if the corresponding enhancers are shared. In this case, correlation alone may fail to identify these ETG pairs.

Based on the predicted ETG pairs, we demonstrated that targets of enhancers are often interspersed by non-targets. We also studied the Hi-C data by Rao *et al*.^12^ and observed that a large fraction of enhancers had their experimentally determined targets interspersed by non-targets of the same enhancers (Supplementary Tables S20 and S21). For instance, in K562, we found that more than 35% of enhancers had interspersed target genes that were supported by more than 20 Hi-C reads. Therefore, the inconsecutiveness of target genes of the same enhancer likely hold for many enhancers.

In the human PETModule model, we used the correlation feature calculated from 13 ENCODE (Encyclopedia of DNA Elements) cell types, which included the three cell types we used to assess the performance of PETModule (MCF7, K562, IMR90). Therefore, the performance of PETModule presented in Table 1 may be biased by the training data. To see the performance of PETModule on independent cell types, we calculated the correlation feature from 12 and 10 ENCODE cell types, respectively, and then tested PETModule on the remaining cell types (Supplementary Table S22). In both cases, we found that the performance of PETModule was highly similar to that presented in Table 1, implying that PETModule may be not biased by the ENCODE data much and will be valuable to predict ETG pairs in diverse cell types generated from projects other than ENCODE.

It is well known that enhancers regulate their targets in a condition-specific way. The current version of PETModule utilizes about a dozen pre-selected conditions to predict ETG pairs. Although its accuracy is comparable with the state-of-the-art computational methods that consider the condition-specific gene expression and others, it is still mandatory to improve PETModule to consider the dynamic regulation of enhancers under specific conditions. Moreover, additional features such as locations of CTCF binding may be considered in the model to take the chromatin boundary into account. We are working on these and other directions to further improve the PETModule tool.

## Methods

### Enhancers and experimentally defined or supported ETG pairs

We downloaded P300 ChIP-seq peaks from 7 human cell lines (MCF7, K562, H1-hESC, HepG2, GM12878, HeLa-S3, and SK-N-SH) from the ENCODE project (https://genome.ucsc.edu/ENCODE/dataMatrix/encodeChipMatrixHuman.html). These cell types were all ENCODE tiers 1 and 2 cell types that had P300 ChIP-seq data. The P300 peaks in a cell type were considered as enhancers in that cell type as previously^18^,^35^. For the human cell line IMR90, we download the known active enhancers from^13^. For mouse data, we used the P300 peaks in the CH12 cell from ENCODE and the RXR-bound enhancers in the macrophage cell from^36^.

We downloaded ETG pairs identified by ChIA-PET in K562 and MCF7 from ref. 25. We downloaded the ETG pairs in IMR90 defined by Jin *et al*. from Hi-C experiments^13^. We also downloaded 387 validated ETG pairs by 3C experiments in mouse macrophage^36^. These ETG pairs were considered as true ETG pairs.

To train PETModule in human, we randomly chose 1000 Hi-C ETG pairs in IMR90 from Jin *et al*.^13^, 1000 ChIA-PET ETG pairs in K562 and 500 ChIA-PET ETG pairs in MCF7 from ref. 25. These pairs were used as positive training data. We also generated 2500 negative ETG pairs, by randomly selecting genes within 2 Mb around enhancers so that the selected genes were not among the known targets of the enhancers. To train PETModule in mouse, we randomly selected 297 ETG pairs from the aforementioned 387 validated pairs as positive data and generated 297 negative ETG pairs similarly as above. All remaining ETG pairs defined by the original studies were then used to test PETModule and other tools.

We also downloaded the normalized 5-kilobase-resolution Hi-C contact matrices in three human cell types (GM12878, IMR90, and K562) and one mouse cell type (CH12) from Rao *et al*.^12^. These matrices described how many Hi-C reads supported the potential interaction of two regions, such as an enhancer region and the promoter region of a gene. One can then use a cutoff or a more complicated strategy to define the interaction of a pair of regions. In this study, we used several cutoffs to define the interacting of a pair of regions to further test PETModule and other tools (Table 2). The defined pairs of interacting regions were then compared with the predicted ETG pairs. A predicted ETG pair was claimed to be supported by Rao *et al*.’s Hi-C data if the enhancer region in this predicted pair overlapped with one region and the promoter region of the gene in this predicted pair overlapped with the other region specified by an interacting pair of regions from the Hi-C contact matrices, under a given cutoff.

### Feature values calculated for enhancer-gene pairs

PETModule considers four features to predict ETG pairs. They are: (1) distance between an enhancer and a gene; (2) CSS of an enhancer and a gene; (3) FSS of an enhancer and a gene; and (4) correlation of DHSs in an enhancer and those in the promoter of a gene. The promoter of a gene is defined as the region from 1 kilobase upstream to 100 bp downstream of its TSS annotated by GENCODE^37^. For simplicity, we called the four features distance, CSS, FSS, and correlation, respectively. For an enhancer and a gene, the distance is calculated as the minimum distance between the endpoints of the enhancer and the TSS of the gene. The correlation is calculated as the Spearman’s rank correlation coefficient of the DHS signals in the enhancer and those in the promoter of the gene. The DHSs across all 13 human ENCODE tiers 1 and 2 cells with DNase-seq data and the DHSs across all 15 mouse ENCODE cell lines with DNase-seq data are used for the correlation calculation^38^. We describe how to calculate CSS and FSS in details in the following.

For an enhancer-gene pair (e, g), the CSS is calculated similarly as that in a previous study^18^ but with the pairwise genome alignments of the reference species and other five vertebrate species. When human enhancers are considered, the five species are chimpanzee, chicken, mouse, frog and zebrafish. When mouse enhancers are considered, the five species are chimpanzee, chicken, human, frog and zebrafish. In brief, we obtain the phylogenetic distances ϕ(r, s) between the reference species r and each of other 5 species^39^. We then calculate the distance *d*_*s*_(*e*, *g*) between the aligned regions of the enhancer and that of the gene in the species s, for each of the other five species. The CSS(e, g) is then calculated by the following formula (1) and (2), with Θ set as 2 Mb:
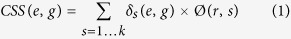

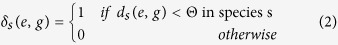


**Figure 2:**
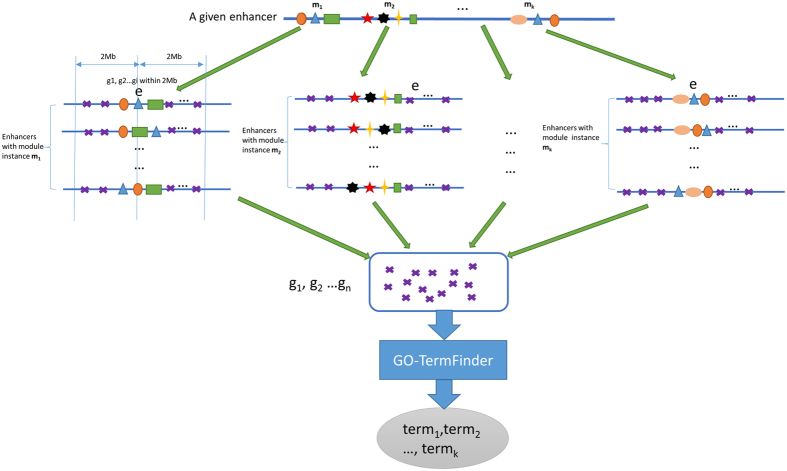
The procedure to calculate the GO terms of an enhancer.

For an enhancer-gene pair (e, g), the FSS measures the GO term similarity of e and g. The GO terms of g can be obtained from http://geneontology.org/page/download-annotations. To define the GO terms of e, one may simply consider the GO terms of each motif with TFBSs in e. However, the number and the annotation of the known motifs are limited. Instead, we run a motif module discovery tool called SIOMICS^40^,^41^ on all enhancer regions under an experimental condition to identify significantly overrepresented motif modules. Recall a motif module is a group of motifs that co-occur in a significant number of genomic regions (enhancers). Because multiple motifs are considered simultaneously by SIOMICS, the random chance for an instance of an identified motif module to occur is much smaller than the random chance for an instance of any identified motif in this motif module to occur. Therefore, the identified motifs by SIOMICS^40^,^41^ are relatively more reliable than the identified motifs by traditional methods considering individual motifs separately^40^,^41^. For each motif module with instances in e, we collect all enhancers containing the instances of this motif module. For each collected enhancer, we collect all genes within 2 Mb of this collected enhancer. We then remove the redundantly collected genes and identify GO terms significantly shared by the remaining collected genes. These GO terms represent the common functions of genes that are likely regulated by motif modules with instances in e, and thus may represent the function of the target genes of e and are considered as the GO terms of e (Fig. 2).

The rationale behind this procedure to obtain the potential GO terms of e is that the function of e may be approximated by motif modules binding to e, and the function of a motif module can be inferred from its target genes. Although we do not know the target genes of motif modules, by the above procedure, the true target genes of motif modules binding to e should be overrepresented in the collected gene list. With the GO terms of e, we calculate the similarity of each GO term of e, say t_1_, and each GO term of g, say t_2_, similarly as in a previous study^18^, by the following formula (3) and (4):
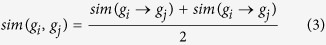

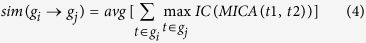
IC is the information content; *MICA* (*t*1, *t*2) is the most informative common ancestor term of t_1_ and t_2_ in the GO annotation dictionary. In this way, we can reasonably approximate the function of an enhancer region and do not depend on the limited knowledge of the occurrence of known motifs in the enhancer region to define its function.

### PETModule, a new approach for predicting ETG pairs based on motif modules

We developed a new approach called PETModule to predict ETG pairs. PETModule applies a random-forests based approach^29^ and is trained with the aforementioned positive and negative ETG pairs. The random forests method is applied as it performs better than the regression based approach such as logistic regression on the data we studied here (Supplementary Table S23). The random forests method builds individual decision trees with a subset of randomly selected training data and then combines all generated trees to classify the testing data. The random forests methods used in PETModule contains five hundred trees. Other number of trees were also evaluated and the random forests based on five hundred trees gave a better ROC AUC and F1 score.

For any input enhancer, PETModule considers all genes within 2 Mb around this enhancer as its potential targets. It then calculates the values of four features (distance, CSS, FSS, correlation) for each potential target. Finally, PETModule applies the trained random-forest based predictor to score a potential target with its four feature values and determine whether this gene is a target of this enhancer. The score calculated by PETModule ranges from 0 to 1, which measures the probability that a potential target is a true target gene of the enhancer under consideration. We choose the probability cutoff of 0.95 as the default cutoff because it gives the best F1 score (Supplementary Table S13).

### Four machine learning approaches for ranking the importance of features

Not all aforementioned four features are the same effective for the ETG pair prediction. To rank those features, we applied the following four machine learning methods: information gain attribute evaluator^42^, SVM^27^, LASSO^28^ and random forests^29^. The information gain attribute evaluator evaluates the contribution of a feature by measuring the information gain with versus without this feature. SVM evaluates the importance of a feature by using an SVM classifier. LASSO constructs a linear model and shrinks the coefficients of non-important features to zero. All features with non-zero regression coefficients are ‘selected’ as important features. The random forests grows many classification trees and assigns a new object to the class most trees vote for. Each of the four methods has been applied to select features in previous studies and demonstrated good performance in feature selection^26^,^30^,^43^,^44^. By applying the four methods to the training data, we ranked the four features used in PETModule.

### Comparison with existing approaches

We compared PETModule with IM-PET^19^ and PreSTIGE^20^. IM-PET requires users to input 1) enhancer positions; 2) genome-wide enhancer signals; and 3) gene expression information to predict ETG pairs. PreSTIGE requires the input of H3K4me1 and RNA-seq data in specified formats for ETG pair prediction. Here we directly used the predicted ETG pairs by authors of IM-PET and PreSTIGE for the common cell types studied by IM-PET, PreSTIGE and PETModule (K562, IMR90, and MCF7).

To calculate the AUC for each tool on a dataset, the following quantities were defined: True Positives (TP), False Positives (FP), False Negatives (FN), and True Negatives (TN). A predicted ETG pair is considered to be a true positive if the region of the enhancer in the predicted pair overlap with one of the genomic regions of the true ETG pair and the promoter region in the predicted pair overlap with the other genomic region of the true ETG pair in the testing data. FP ETG pairs are the predicted ETG pairs not supported by the true ETG pairs in the testing data. FN ETG pairs are those not predicted true ETG pairs and TN ETG pairs are those not predicted ETG pairs that are specified in the negative testing datasets. The true positive rate (TPR) is then defined as TP/(TP+FN) and the false positive rate (FPR) is defined as FP/(FP+TN). The ROC curve is then generated with the calculated TPR and FPR values derived with different classifier decision thresholds. The AUC is calculated as the area under this ROC curve.

## Additional Information

**How to cite this article**: Zhao, C. *et al*. PETModule: a motif module based approach for enhancer target gene prediction. *Sci. Rep.* **6**, 30043; doi: 10.1038/srep30043 (2016).

## Supplementary Material

Supplementary Information

Supplementary Tables

## Figures and Tables

**Table 1:** PETModule prediction on three datasets with experimentally defined ETG pairs.

Dataset	Enhancers	Known pairs	Predicted pairs	Known pairs predicted	Recall	Precision	ROC AUC	F1 score
ChIA-PET (K562)	3300	4110	9244	1917	0.466	0.207	0.938	0.287
ChIA-PET (MCF7)	341	370	560	187	0.505	0.334	0.968	0.402
Hi-C (IMR90)	10920	19666	26467	7811	0.397	0.295	0.942	0.338
Overall	14561	24146	36271	9915	0.411	0.273	0.949	0.328

The known ETG pairs here do not contain any of the positive ETG pairs used for training.

**Table 2:** PETModule prediction on IMR90 assessed with Hi-C contact matrices.

Cutoff	#Enhancers with supporting Hi-C data	#Predicted ETG pairs	#Known ETG pairs	#Known ETG pairs predicted	Recall	Precision	ROC AUC	F1 score
5	10881	23454	64075	17354	0.271	0.740	0.890	0.397
10	9918	22869	32837	12031	0.366	0.526	0.914	0.432
15	8433	21145	20319	8413	0.414	0.398	0.924	0.406
20	7069	19131	14024	6054	0.431	0.316	0.928	0.365
25	5945	17025	10219	4479	0.438	0.263	0.929	0.329

The cutoff specifies the minimum number of supporting Hi-C reads required to define known ETG pairs. The known ETG pairs here do not contain any of the positive ETG pairs used for training.

**Table 3:** Prediction results on two mouse cells.

Prediction Model	Dataset	Enhancers	Known pairs	Predicted pairs	Known pairs predicted	Recall	Precision	ROC AUC	F1 score
Human model	CH12	14195	24516	124102	16540	0.667	0.133	0.938	0.220
	macrophage	387	387	3171	251	0.650	0.076	0.923	0.135
Mouse model	CH12	14195	24516	64512	18252	0.744	0.283	0.968	0.410
	macrophage	387	387	1468	271	0.700	0.167	0.961	0.269

**Table 4:** Comparison of PETModule with IM-PET and PreSTIGE.

Dataset	Tools	Enhancers	Known pairs	Predicted pairs	Known pairs predicted	Recall	Precision	ROC AUC	F1 score
ChIA-PET (K562)	PETModule	694	907	2285	429	0.473	0.188	0.938	0.269
	IM-PET	694	907	1872	278	0.307	0.149	0.88	0.200
	PreSTIGE	694	907	1468	382	0.421	0.260	0.8	0.322
ChIA-PET (MCF7)	PETModule	94	107	282	61	0.570	0.216	0.968	0.314
	IM-PET	94	107	191	33	0.308	0.173	0.88	0.221
	PreSTIGE	94	107	178	62	0.579	0.348	0.8	0.435
Hi-C (IMR90)	PETModule	202	411	714	184	0.448	0.258	0.942	0.327
	IM-PET	202	411	282	75	0.182	0.266	0.89	0.216
	PreSTIGE	202	411	342	114	0.277	0.333	0.8	0.303
Overall	PETModule	990	1425	3281	674	0.473	0.205	0.949	0.286
	IM-PET	990	1425	2345	386	0.271	0.164	0.88	0.205
	PreSTIGE	990	1425	1988	558	0.392	0.281	0.8	0.327

Only the common enhancers with predictions by three methods were considered.

## References

[b1] Blackwood E. M. & Kadonaga J. T. Going the distance: a current view of enhancer action. Science 281, 60–63 (1998). 9679020 10.1126/science.281.5373.60

[b2] Pennacchio L. A., Bickmore W., Dean A., Nobrega M. A. & Bejerano G. Enhancers: five essential questions. Nature reviews Genetics 14, 288–295, doi: 10.1038/nrg3458 (2013). PMC4445073 23503198

[b3] Maston G. A., Evans S. K. & Green M. R. Transcriptional regulatory elements in the human genome. Annu. Rev. Genomics Hum. Genet. 7, 29–59 (2006). 16719718 10.1146/annurev.genom.7.080505.115623

[b4] Latchman D. S. Transcription factors: an overview. The international journal of biochemistry & cell biology 29, 1305–1312 (1997). 9570129 10.1016/s1357-2725(97)00085-x

[b5] Lenhard B. & Wasserman W. W. TFBS: Computational framework for transcription factor binding site analysis. Bioinformatics 18, 1135–1136 (2002). 12176838 10.1093/bioinformatics/18.8.1135

[b6] van Arensbergen J., van Steensel B. & Bussemaker H. J. In search of the determinants of enhancer–promoter interaction specificity. Trends in cell biology 24, 695–702 (2014). 25160912 10.1016/j.tcb.2014.07.004 PMC4252644

[b7] Dekker J. The three’C’s of chromosome conformation capture: controls, controls, controls. Nature methods 3, 17–21 (2006). 16369547 10.1038/nmeth823

[b8] Simonis M. . Nuclear organization of active and inactive chromatin domains uncovered by chromosome conformation capture–on-chip (4C). Nature genetics 38, 1348–1354 (2006). 17033623 10.1038/ng1896

[b9] Dostie J. . Chromosome Conformation Capture Carbon Copy (5C): a massively parallel solution for mapping interactions between genomic elements. Genome research 16, 1299–1309 (2006). 16954542 10.1101/gr.5571506 PMC1581439

[b10] Belton J.-M. . Hi–C: a comprehensive technique to capture the conformation of genomes. Methods 58, 268–276 (2012). 22652625 10.1016/j.ymeth.2012.05.001 PMC3874846

[b11] Lieberman-Aiden E. . Comprehensive mapping of long-range interactions reveals folding principles of the human genome. Science 326, 289–293 (2009). 19815776 10.1126/science.1181369 PMC2858594

[b12] Rao S. S. P. . A 3D Map of the Human Genome at Kilobase Resolution Reveals Principles of Chromatin Looping. Cell 159, 1665–1680, doi: 10.1016/j.cell.2014.11.021 (2014). 25497547 PMC5635824

[b13] Jin F. . A high-resolution map of the three-dimensional chromatin interactome in human cells. Nature 503, 290–294 (2013). 24141950 10.1038/nature12644 PMC3838900

[b14] Fullwood M. J., Han Y., Wei C. L., Ruan X. & Ruan Y. Chromatin interaction analysis using paired‐end tag sequencing. Current Protocols in Molecular Biology , 21.15. 21–21.15. 25 (2010). 10.1002/0471142727.mb2115s89 PMC6924956 20069536

[b15] Sanyal A., Lajoie B. R., Jain G. & Dekker J. The long-range interaction landscape of gene promoters. Nature 489, 109–113 (2012). 22955621 10.1038/nature11279 PMC3555147

[b16] Thurman R. E. . The accessible chromatin landscape of the human genome. Nature 489, 75–82 (2012). 22955617 10.1038/nature11232 PMC3721348

[b17] Shen Y. . A map of the cis-regulatory sequences in the mouse genome. Nature 488, 116–120 (2012). 22763441 10.1038/nature11243 PMC4041622

[b18] Rodelsperger C. . Integrative analysis of genomic, functional and protein interaction data predicts long-range enhancer-target gene interactions. Nucleic acids research 39, 2492–2502, doi: 10.1093/nar/gkq1081 (2011). 21109530 PMC3074119

[b19] He B., Chen C., Teng L. & Tan K. Global view of enhancer–promoter interactome in human cells. Proceedings of the National Academy of Sciences 111, E2191–E2199 (2014). 10.1073/pnas.1320308111 PMC4040567 24821768

[b20] Corradin O. . Combinatorial effects of multiple enhancer variants in linkage disequilibrium dictate levels of gene expression to confer susceptibility to common traits. Genome research 24, 1–13 (2014). 24196873 10.1101/gr.164079.113 PMC3875850

[b21] Zhang T. On the consistency of feature selection using greedy least squares regression. JMLR - Journal of Machine Learning Research 10, 555–568 (2009).

[b22] Cai X. . Systematic identification of conserved motif modules in the human genome. BMC genomics 11, 567, doi: 10.1186/1471-2164-11-567 (2010). 20946653 PMC3091716

[b23] Ding J., Cai X., Wang Y., Hu H. & Li X. ChIPModule: systematic discovery of transcription factors and their cofactors from ChIP-seq data. Pacific Symposium on Biocomputing. Pacific Symposium on Biocomputing 18, 320–331 (2013). 23424137

[b24] Hu J., Hu H. & Li X. MOPAT: a graph-based method to predict recurrent cis-regulatory modules from known motifs. Nucleic acids research 36, 4488–4497, doi: 10.1093/nar/gkn407 (2008). 18606616 PMC2490743

[b25] Li G. . Extensive promoter-centered chromatin interactions provide a topological basis for transcription regulation. Cell 148, 84–98 (2012). 22265404 10.1016/j.cell.2011.12.014 PMC3339270

[b26] Lee C. & Lee G. G. Information gain and divergence-based feature selection for machine learning-based text categorization. Information processing & management 42, 155–165 (2006).

[b27] Suykens J. A. & Vandewalle J. Least squares support vector machine classifiers. Neural processing letters 9, 293–300 (1999).

[b28] Tibshirani R. Regression shrinkage and selection via the lasso. *Journal of the Royal Statistical Society. Series B* (*Methodological*) 58, 267–288 (1996).

[b29] Liaw A. & Wiener M. Classification and regression by randomForest. R news 2, 18–22 (2002).

[b30] Saeys Y., Abeel T. & Van de Peer Y. In Machine learning and knowledge discovery in databases 313–325 (Springer, 2008).

[b31] Chen Y.-W. & Lin C.-J. In Feature extraction 315–324 (Springer, 2006).

[b32] Saeys Y., Inza I. & Larrañaga P. A review of feature selection techniques in bioinformatics. Bioinformatics 23, 2507–2517 (2007). 17720704 10.1093/bioinformatics/btm344

[b33] Blanchette M. . Genome-wide computational prediction of transcriptional regulatory modules reveals new insights into human gene expression. Genome research 16, 656–668, doi: 10.1101/gr.4866006 (2006). 16606704 PMC1457048

[b34] Ding J., Hu H. & Li X. Thousands of cis-regulatory sequence combinations are shared by Arabidopsis and poplar. Plant physiology 158, 145–155, doi: 10.1104/pp.111.186080 (2012). 22058225 PMC3252106

[b35] Visel A. . ChIP-seq accurately predicts tissue-specific activity of enhancers. Nature 457, 854–858 (2009). 19212405 10.1038/nature07730 PMC2745234

[b36] Daniel B. . The active enhancer network operated by liganded RXR supports angiogenic activity in macrophages. Genes & development 28, 1562–1577 (2014). 25030696 10.1101/gad.242685.114 PMC4102764

[b37] Harrow J. . GENCODE: the reference human genome annotation for The ENCODE Project. Genome research 22, 1760–1774 (2012). 22955987 10.1101/gr.135350.111 PMC3431492

[b38] Maurano M. T. . Systematic localization of common disease-associated variation in regulatory DNA. Science 337, 1190–1195 (2012). 22955828 10.1126/science.1222794 PMC3771521

[b39] Miller W. . 28-way vertebrate alignment and conservation track in the UCSC Genome Browser. Genome research 17, 1797–1808 (2007). 17984227 10.1101/gr.6761107 PMC2099589

[b40] Ding J., Dhillon V., Li X. & Hu H. Systematic discovery of cofactor motifs from ChIP-seq data by SIOMICS. Methods 79–80, 47–51, doi: 10.1016/j.ymeth.2014.08.006 (2015). 25171961

[b41] Ding J., Hu H. & Li X. SIOMICS: a novel approach for systematic identification of motifs in ChIP-seq data. Nucleic acids research 42, e35, doi: 10.1093/nar/gkt1288 (2014). 24322294 PMC3950686

[b42] Kira K. & Rendell L. A. In Proceedings of the ninth international workshop on Machine learning 249–256 (1992).

[b43] Chang Y.-W. & Lin C.-J. Feature ranking using linear svm. Causation and Prediction Challenge Challenges in Machine Learning 2, 47 (2008).

[b44] Ghaoui L. E., Viallon V. & Rabbani T. Safe feature elimination for the lasso and sparse supervised learning problems. *arXiv preprint arXiv:1009.4219* (2010).

