# Supplementary material for: PETModule: a motif module based approach for enhancer target gene prediction
Source: Sci Rep. 2016 Jul 20;6:30043. doi: 10.1038/srep30043 (PMC4951774; doi:10.1038/srep30043)
Supplement: Supplementary Information [file srep30043-s1.pdf]

PETModule: a motif module based approach for enhancer target gene prediction

Changyong Zhao[1], Xiaoman Li[2, §], Haiyan Hu[1, §]

[1]Department of Electrical Engineering & Computer Science, University of Central Florida, Orlando, FL, 32816, USA

[2]Burnett School of Biomedical Science, University of Central Florida, Orlando, FL, 32816, USA

[§]Corresponding authors

Email addresses:

CZ: cyzhao@knights.ucf.edu

XL: xiaoman@mail.ucf.edu

HH: haihu@cs.ucf.edu

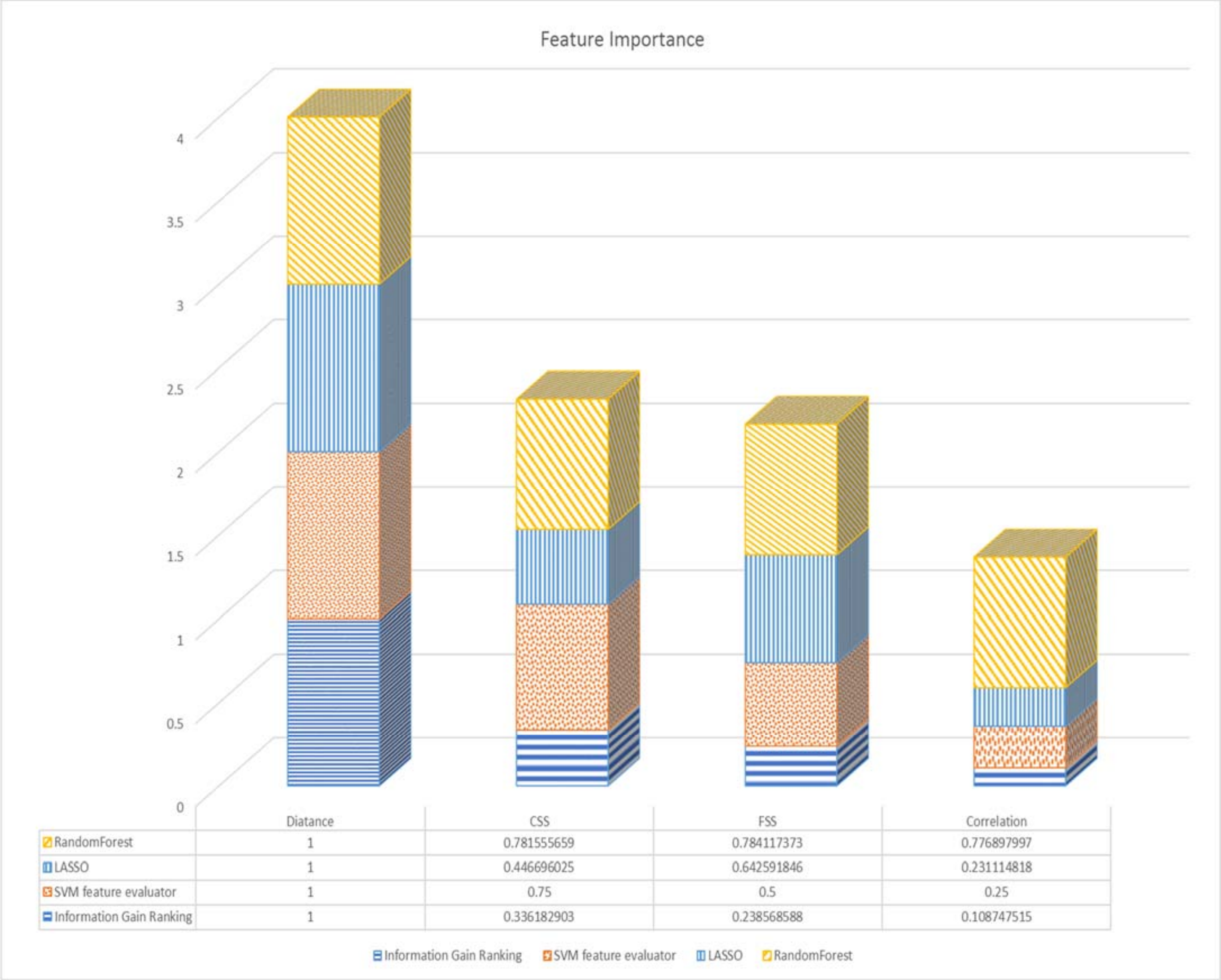

Figure S1. Feature ranking. The correlation score is calculated by using 12 cells or tissues type as used in PreSTIGE.

Table S1. Prediction performance of PETModule on K562 using the normalized Hi-C contact matrices.

Cutoff	#Enhancers with supporting Hi-C data	#predicted ETG pairs that are at least 5kb away from each other	#known pairs	#known pairs predicted	recall	precision	F1 score	ROC AUC
5	3923	10001	32242	7402	0.230	0.740	0.350	0.894
10	3485	9502	14887	4574	0.307	0.481	0.375	0.913
15	2957	8591	8730	3011	0.344	0.350	0.348	0.916
20	2428	7556	5907	2052	0.347	0.271	0.305	0.915
25	2015	6583	4300	1429	0.332	0.217	0.263	0.909

The cutoff in the first column specifies the minimum number of supporting Hi-C reads required to define ETG pairs. The known ETG pairs here do not contain any of the positive ETG pairs used for training.

Table S2. Prediction performance of PETModule on GM12878 using the normalized Hi-C contact matrices.

Cutoff	#Enhancers with supporting Hi-C data	#predicted ETG pairs that are at least 5kb away from each other	#known pairs	#known pairs predicted	recall	precision	F1 score	ROC AUC
5	6186	11117	102504	10649	0.104	0.958	0.187	0.816
10	6126	11116	59898	10083	0.168	0.907	0.284	0.867
15	6035	11099	42865	9282	0.217	0.836	0.344	0.888
20	5887	11059	33444	8548	0.256	0.773	0.384	0.899
25	5693	10987	27142	7814	0.288	0.711	0.410	0.906

The cutoff in the first column specifies the minimum number of supporting Hi-C reads required to define known ETG pairs. The known ETG pairs do not contain any of the positive ETG pairs used for training.

Table S3. The percentage of the predicted ETG pairs in K562 support by Hi-C contact matrices.

The minimum number of Hi-C reads required to define a ETG pair	5	10	15	20	25
%Predicted ETG pairs in 2Mb supported	0.740	0.481	0.350	0.271	0.217
%Predicted ETG pairs in 1Mb supported	0.710	0.460	0.330	0.264	0.223
%Random ETGs pairs in 1mb supported	0.190	0.095	0.064	0.048	0.043
%Random ETGs pairs in 2mb supported	0.102	0.053	0.036	0.029	0.026

The fraction in a cell is the percentage of the ETG pairs supported by at least the given number of Hi-C reads. We required different minimum number of supporting Hi-C reads from 5 to 25.

Table S4. The percentage of the predicted ETG pairs in GM12878 support by Hi-C contact matrices.

The minimum number of Hi-C reads required to define a ETG pair	5	10	15	20	25
%Predicted ETG pairs in 2Mb supported	0.958	0.907	0.836	0.773	0.711
%Predicted ETG pairs in 1Mb supported	0.950	0.905	0.839	0.769	0.706
%Random ETGs pairs in 1mb supported	0.537	0.330	0.237	0.186	0.153
%Random ETGs pairs in 2mb supported	0.309	0.186	0.131	0.102	0.085

The fraction in a cell is the percentage of the ETG pairs supported by at least the given number of Hi-C reads. We required different minimum number of supporting Hi-C reads from 5 to 25.

Table S5. The percentage of the predicted ETG pairs in IMR90 support by Hi-C contact matrices.

The minimum number of Hi-C reads required to define a ETG pair	5	10	15	20	25
%Predicted ETG pairs in 2Mb supported	0.740	0.526	0.398	0.316	0.263
%Predicted ETG pairs in 1Mb supported	0.717	0.501	0.377	0.302	0.258
%Random ETGs pairs in 1mb supported	0.190	0.101	0.071	0.058	0.049
%Random ETGs pairs in 2mb supported	0.102	0.054	0.038	0.031	0.026

The fraction in a cell is the percentage of the ETG pairs supported by at least the given number of Hi-C reads. We required different minimum number of supporting Hi-C reads from 5 to 25.

Table S6. Significance of predicted ETGs in K562 supported by the Hi-C contact matrices.

Cutoff	#predicted ETG pairs	#predicted ETG pairs supported	%Random ETG pairs supported	P-value
5	10001	7402	0.102	0
10	9502	4574	0.053	0
15	8591	3011	0.036	0
20	7556	2052	0.029	0
25	6583	1429	0.026	0

The p-values measure the binomial tail probability of observing at least the observed number of predicted ETG pairs supported under different cutoffs.

Table S7. Significance of predicted ETGs in GM12878 supported by the Hi-C contact matrices.

Cutoff	#predicted ETG pairs	#predicted ETG pairs supported	%Random ETG pairs supported	P-value
5	11117	10649	0.309	0
10	11116	10083	0.186	0
15	11099	9282	0.131	0
20	11059	8548	0.102	0
25	10987	7814	0.085	0

The p-values measure the binomial tail probability of observing at least the observed number of predicted ETG pairs supported under different cutoffs.

Table S8. Significance of predicted ETGs in IMR90 supported by the Hi-C contact matrices.

Cutoff	#predicted ETG pairs	#predicted ETG pairs supported	%Random ETG pairs supported	P-value
5	23454	17354	0.102	0
10	22869	12031	0.054	0
15	21145	8413	0.038	0
20	19131	6054	0.031	0
25	17025	4479	0.026	0

The p-values measure the binomial tail probability of observing at least the observed number of predicted ETG pairs supported under different cutoffs.

Table S9. Prediction performance comparison between PETModule in 1Mb and PETModule in 2Mb

DataSet	PETModule Predict range	enhancers	known pairs	predicted pairs	known pairs predicted	recall	precision	ROC AUC	F1 score
ChIA-PET (K562)	1Mb	4080	5110	4111	1010	0.198	0.246	0.937	0.219
	2Mb	4080	5110	11532	2382	0.466	0.207	0.938	0.286
ChIA-PET (MCF7)	1Mb	790	870	641	284	0.326	0.443	0.911	0.376
	2Mb	790	870	1709	477	0.548	0.279	0.968	0.370
Hi-C (IMR90)	1Mb	11497	20666	9762	3832	0.185	0.393	0.905	0.252
	2Mb	11497	20666	27468	8123	0.393	0.296	0.942	0.338
Overall	1Mb	16367	26646	14514	5126	0.192	0.353	0.918	0.249
	2Mb	16367	26646	40709	10982	0.412	0.270	0.949	0.326

Table S10. Inconsecutiveness of targets of enhancers in the genome

cell	inconsecutive rate
A549	0.705
GM12878	0.685
Helas3	0.671
hEsc	0.745
IMR90	0.735
k562	0.746
MCF7	0.666
SK-N-SH	0.640
Average	0.699

Prediction performance using old version GOA

We also made analysis whether the version of GOA influence our prediction model. We download GOA annotation file 2010 version from UniProt-GOA. Then we used the old version GOA annotation to calculate the GO similarity score. After that we build the matrix and did the model train and test data using IMR90 enhancer data to test the model. Following is the performance difference between old version and new version GOA annotation file. From the table we can see that prediction model with new version GOA have a better performance.

Table S11. Performance comparison between models with new version GOA and old version GOA

Performance of classifier	TP Rate	FP Rate	Precision	Recall	F1 score	ROC Area
Random Forest(using new version GOA)	0.805	0.148	0.203	0.805	0.324	0.903
Random Forest(using 2010 version GOA)	0.792	0.145	0.203	0.792	0.323	0.901
Random Forest(using 2005 version GOA)	0.711	0.122	0.213	0.711	0.328	0.89
Random Forest(using 2001 version GOA)	0.681	0.115	0.216	0.681	0.328	0.885

Table S12 Comparison details of three enhancer target prediction tools in three dataset.

DataSet	Tools	enhancers	known pairs	predicted pairs	known pairs predicted	recall	precision	ROC AUC	F1 score
ChIA-PET (K562)	PETModule	3300	4110	9244	1917	0.466	0.207	0.938	0.287
	IM-PET	1905	2469	3204	618	0.25	0.193	0.88	0.218
	PreSTIGE	614	785	1030	302	0.385	0.293	0.8	0.333
ChIA-PET (MCF7)	PETModule	341	370	1709	477	0.505	0.334	0.968	0.402
	IM-PET	269	313	413	88	0.281	0.213	0.88	0.242
	PreSTIGE	192	223	338	137	0.614	0.405	0.8	0.488
Hi-C (IMR90)	PETModule	10920	19666	26467	7811	0.397	0.295	0.942	0.338
	IM-PET	2387	4584	2826	850	0.185	0.3	0.89	0.229
	PreSTIGE	686	1385	1110	404	0.292	0.364	0.8	0.324
Overall	PETModule	14561	24146	36271	9915	0.411	0.273	0.949	0.328
	IM-PET	4561	7366	6443	1556	0.211	0.241	0.883	0.225
	PreSTIGE	1492	2393	2478	843	0.352	0.340	0.8	0.346

Table S13. Performance of PETModule with different probability cutoffs to define ETGs.

cutoff	recall	precision	F1 score
0.7	0.754	0.164	0.269
0.75	0.719	0.176	0.283
0.8	0.676	0.191	0.297
0.85	0.619	0.209	0.313
0.9	0.536	0.232	0.324
0.95	0.412	0.270	0.326
0.96	0.377	0.280	0.321
0.97	0.337	0.292	0.313
0.98	0.293	0.309	0.301
0.99	0.235	0.328	0.274
1.0	0.158	0.357	0.219

We Use Rao’s Hic matrix data to compare PETModule and IM-PET in three cell lines-K562, IMR90, GM12878. Our methods show a better performance on precision, recall and F score in three cell lines.

Table S14. Prediction performance comparison in K562 cell

Contacts Reads cutoff	tools	recall	Precision	F1 score
5	PETModule	0.230	0.740	0.350
	IM-PET	0.092	0.513	0.156
	PreSTIGE	0.169	0.745	0.276
10	PETModule	0.307	0.481	0.375
	IM-PET	0.136	0.391	0.202
	PreSTIGE	0.243	0.543	0.336
15	PETModule	0.345	0.350	0.348
	IM-PET	0.168	0.332	0.223
	PreSTIGE	0.268	0.410	0.325
20	PETModule	0.347	0.272	0.305
	IM-PET	0.188	0.293	0.229
	PreSTIGE	0.279	0.332	0.303
25	PETModule	0.332	0.217	0.263
	IM-PET	0.199	0.261	0.226
	PreSTIGE	0.275	0.276	0.275

Table S15. Prediction performance comparison in IMR90

Contacts Reads cutoff	tool	recall	Precision	F1 score
5	PETModule	0.271	0.740	0.397
	IM-PET	0.081	0.536	0.142
	PreSTIGE	0.151	0.752	0.252
10	PETModule	0.366	0.526	0.432
	IM-PET	0.124	0.450	0.195
	PreSTIGE	0.210	0.550	0.303
15	PETModule	0.414	0.398	0.406
	IM-PET	0.158	0.408	0.228
	PreSTIGE	0.201	0.413	0.296
20	PETModule	0.432	0.316	0.365
	IM-PET	0.185	0.381	0.249
	PreSTIGE	0.239	0.331	0.278
25	PETModule	0.438	0.263	0.329
	IM-PET	0.204	0.354	0.259
	PreSTIGE	0.246	0.285	0.264

Table S16. Prediction performance comparison in GM12878

Contacts Reads cutoff	tool	recall	Precision	F1 score
5	PETModule	0.104	0.958	0.187
	IM-PET	0.041	0.768	0.078
	PreSTIGE	0.062	0.907	0.117
10	PETModule	0.168	0.907	0.284
	IM-PET	0.059	0.663	0.108
	PreSTIGE	0.103	0.889	0.185
15	PETModule	0.216	0.826	0.344
	IM-PET	0.072	0.598	0.129
	PreSTIGE	0.137	0.859	0.237
20	PETModule	0.256	0.773	0.384
	IM-PET	0.083	0.547	0.145
	PreSTIGE	0.167	0.818	0.278
25	PETModule	0.289	0.711	0.410
	IM-PET	0.094	0.508	0.158
	PreSTIGE	0.191	0.768	0.306

Table S17. Running time comparison with IM-PET

Tools	enhancer Number	running time
PETModule	5000	about 5 hours
IM-PET	5000	30 minutes
PreSTIGE	5000	6 hours

Table S18. Predicted ETG pairs were shared across cells.

Cells	Enhancers that have predicted targets	ETG pairs predicted	enhancers shared by at least one of other 7 cells	Shared ETG pairs when enhancers shared	ETG pairs not shared when enhancers were shared
A549	13065	40135	7080 (54.9%)	16618 (75.3%)	5442 (24.7%)
K562	3635	11765	1188 (32.7%)	2932 (72.9%)	1089 (27.1%)
MCF7	635	1750	318(50.1%)	667(72.3%)	399(27.9%)
Gm12878	4545	12657	858 (18.9%)	1930 (74.0%)	679 (26.0%)
Helas3	18083	46603	6884 (38.1%)	14393 (74.1%)	5027 (25.9%)
hEsc	7222	29678	1798 (24.9%)	6196 (72.5%)	2352 (27.5%)
IMR90	9302	27853	2786 (30.0%)	5892 (68.3%)	2737 (31.7%)
SK-N-SH	10962	25622	1720 (15.7%)	3336 (70.1%)	1422 (29.9%)
Average	8431	24508	2829(33.6%)	6496(73.1%)	2393(26.9%)

Table S19. Experimentally supported ETG pairs were shared across cells.

Cutoff	Cells	Enhancers that have targets	enhancers shared by the other cell	Shared ETG pairs when enhancers were shared	ETG pairs not shared when enhancers shared
15	K562	2957	207	504(82.0%)	111(18.0%)
	IMR90	8433	193	468(71.1%)	200(29.9%)
20	K562	2428	167	302(81.6%)	68(18.4%)
	IMR90	7069	156	288(65.6%)	151(34.4%)
25	K562	2015	128	220(85.3%)	38(14.7%)
	IMR90	5945	119	205(72.2%)	79(27.8%)

The cutoff specifies the minimum number of supporting Hi-C reads required to define ETG pairs.

Table S20. Inconsecutiveness of ETG pairs supported by Rao’s Hi-C data in the genome (IMR90)

Cutoff	inconsecutive rate
5	0.659038691297
10	0.425186529542
15	0.309616980908
20	0.260574338662
25	0.229436501262

The cutoff specifies the minimum number of supporting Hi-C reads required to define ETG pairs.

Table S21. Inconsecutiveness of ETG pairs supported by Rao’s Hi-C data in the genome (K562)

Cutoff	inconsecutive rate
5	0.707366811114
10	0.471736011478
15	0.393642204937
20	0.368204283361
25	0.357816377171

The cutoff specifies the minimum number of supporting Hi-C reads required to define ETG pairs.

Table S22. The performance comparison of PETModule based on correlation calculated from 13, 12 and 10 ENCODE cell types.

Dataset	Pre-selected cell Information	enhancers	known pairs	predicted pairs	known pairs predicted	recall	precision	ROC AUC	F1 score
ChIA-PET(K562)	13 cell types	3300	4110	9244	1917	0.466	0.207	0.938	0.287
	12 cell types(No K562)	3300	4110	9075	1820	0.443	0.201	0.934	0.276
	10 cell types	3300	4110	9083	1824	0.444	0.201	0.934	0.277
ChIA-PET(MCF7)	13 cell types	341	370	560	187	0.505	0.334	0.968	0.402
	12 cell types (No MCF7)	341	370	560	187	0.505	0.334	0.96	0.402
	10 cell types	341	370	543	174	0.47	0.32	0.956	0.38
Hi-C(IMR90)	13 cell types	10920	19666	26467	7811	0.397	0.295	0.942	0.338
	12 cell types (No IMR90)	10920	19666	26323	7713	0.382	0.293	0.94	0.335
	10 cell types	10920	19666	26671	7685	0.391	0.288	0.94	0.332
Overall	13 cell types	14561	24146	36271	9915	0.411	0.273	0.949	0.328
	12 cell types	14561	24146	35958	9720	0.403	0.270	0.945	0.323
	10 cell types	14561	24146	36297	9683	0.401	0.268	0.943	0.321

For each cell type in the first column, the first row is the default PETModule performance; the second row is the cross-validation performance of PETModule in the corresponding cell type; the third row is the performance of PETModule with the correlation calculated from the remaining 10 cell types (without K562, MCF7, and IMR90).

Table S23. Comparison of random forest based approach and logistic regression based approach.

Performance of classifier	TP Rate	FP Rate	Precision	Recall	F1 score	ROC AUC
Logistic regression	0.833	0.167	0.835	0.833	0.832	0.906
Random Forest	0.837	0.164	0.837	0.837	0.836	0.915
